# Age-associated clinical characteristics and *ATP7B* mutation landscape in pediatric Wilson’s disease: a study from southwest China

**DOI:** 10.3389/fgene.2026.1839993

**Published:** 2026-06-16

**Authors:** Tingting Wu, Yuanzhen Zhang, Xiaorong Peng, Yunnan Chang, Tao Qin, Jing Zhu, Qiqin Tang, Jiao Tian, Ruiqiu Zhao, Hongmei Xu

**Affiliations:** Department of Infectious Diseases, Children’s Hospital of Chongqing Medical University, National Clinical Research Center for Children and Adolescents’ Health and Diseases, Ministry of Education Key Laboratory of Child Development and Disorders, Chongqing Key Laboratory of Child Rare Diseases in Infection and Immunity, Chongqing, China

**Keywords:** *ATP7B*, genotype-phenotype correlation, mutational spectrum, pediatric, Wilson’s disease

## Abstract

**Background:**

Wilson’s disease (WD) is a rare autosomal recessive disorder of copper metabolism caused by pathogenic variants in *ATP7B*. However, the regional mutational spectrum, genotype–phenotype correlations, and biochemical trajectories in pediatric populations from Southwest China remain incompletely defined.

**Methods:**

In total, 170 pediatric patients with WD were enrolled. The clinical data, imaging findings, and biochemical parameters were obtained. Age-stratified analyses were also conducted. *ATP7B* variants were analyzed in 81 patients using whole-gene sequencing. Genotype–phenotype associations were evaluated using logistic regression.

**Results:**

Hepatic presentation was predominant (84.12%), with neurological and mixed phenotypes more common in older patients. The incidence of Kayser-Fleischer rings, hepatic steatosis, cirrhosis, and abnormal brain magnetic resonance imaging (MRI) findings increased with age at diagnosis (all p < 0.01). Protein-truncating variants were significantly associated with neurological involvement (odds ratio [OR] = 4.058, p = 0.01), and male sex was an independent predictor (OR = 5.342, p = 0.006). The p. Pro992Leu variant was associated with hepatic fibrosis and steatosis (both p = 0.001). Liver enzymes (alanine aminotransferase [ALT], aspartate aminotransferase [AST], and gamma-glutamyltransferase [GGT]) decreased with increasing age at onset, whereas total cholesterol and low-density lipoprotein [LDL] cholesterol levels increased. 76 *ATP7B* variants were identified, with exon 8 being the primary hotspot. The most frequent alleles were c.2333G>T, c.2975C>T, and c.2310C>G. Co-occurrence analysis identified c.2975C>T as the central allele, with extensive allelic heterogeneity observed.

**Conclusion:**

This study aimed to investigate age-related differences in clinical and biochemical features at disease onset in pediatric WD, with complementary analyses of genetic variation. It delineates age of onset-dependent phenotypic progression and a highly heterogeneous *ATP7B* mutational landscape in pediatric WD. Intronic variants and rare insertions further expand the mutation spectrum.

## Introduction

Wilson disease (WD) is an autosomal recessive disorder of copper metabolism caused by mutations in a gene encoding the copper transporter p-type ATPase (*ATP7B*) (Członkowska et al., 2018; Strazzullo et al., 2003). *ATP7B* is located on the short arm of chromosome 13, and the proteins it encodes are responsible for the translocation of intracellularly bound copper into the secretory pathway, where excess copper is subsequently excreted into bile and incorporated into apoceruloplasmin to synthesize functional ceruloplasmin (Strazzullo et al., 2003; Petrukhin et al., 1993). The toxic deposition of copper throughout the body results in highly heterogeneous clinical presentations, including hepatic impairment, neurological disturbances, and other systemic derangements (Członkowska et al., 2018; Petrukhin et al., 1993; Ferenci, 2006). WD predominantly manifests as progressive liver injury. As the central organ of lipid metabolism, the liver processes triglycerides, cholesterol, phospholipids, and glycolipids, and simultaneously serves as a major reservoir and metabolic site for copper ions (Fontes et al., 2024; Blades et al., 2021). The disruption of copper homeostasis leads to toxic hepatic copper overload, which in turn impairs lipid synthesis and transport (Blades et al., 2021). Abnormal lipid accumulation of lipids triggers a cascade of organelle dysfunction, inflammation, and fibrogenesis, ultimately activating hepatic stellate cells and promoting the progression of fibrosis to cirrhosis, the principal cause of death in patients with WD (Langner and Denk, 2004). The relationship between hepatic steatosis and WD, and the potential utility of hepatic steatosis as an early diagnostic marker of WD, warrants further investigation. WD can develop at any age, usually between the ages of 5 and 35 years, and with the widespread use of genetic testing, it is increasingly being diagnosed in children younger than 5 years of age, and the clinical presentation in younger children may be nonspecific (Roberts and Schilsky, 2023). Clinical diagnosis may be more difficult in children with asymptomatic WD.

Therefore, if more accurate diagnostic approaches are to be used, genetic testing will become increasingly important for the early, rapid, and accurate diagnosis of WD (Członkowska et al., 2018). *ATP7B* was localized to chromosome 13q14 in 1993, it consists of 20 introns and 21 exons (Yang et al., 2023). Double-allele mutations in *ATP7B* cause WD. Studies have shown that mutations affecting key parts of the protein, including the copper-binding structural domain or the ATPase loop, may contribute to the early onset of liver disease. This gene encodes the copper-transporting ATPase (Cu -ATPase) (Schilsky et al., 2023), a transmembrane protein that transports copper using ATP hydrolysis energy. Cu- ATPase has several functional domains: the copper-binding domain regulates cellulase activity, the transmembrane structural domain forms the Cu-channel pore, the ATP-binding domain has a distinctive nucleotide-liganding environment (consisting of two parts: the p-structural domain and the p-type atpase and the n-structural domain with the characteristic motifs), and the a structural domain is essential for the enzymatic function of p-type ATPases (Yang et al., 2023). According to the Human Gene Mutation Database and comprehensive variant compilations, more than 1,000 disease-causing variants of the *ATP7B* variants have been identified in patients with WD to date (Beyzaei et al., 2024). More than 700 *ATP7B* variants have been identified to date. The most common variant in Central, Eastern and Northern European populations is the point variant H1069Q (exon 14), whereas in Asia, it is R778L (exon 8), with allele frequencies ranging from 28.4% to 49.2% in WD patients (Ferenci, 2006; Beyzaei et al., 2024). More than 50% of the variants are missense and nonsense, and the rest may be insertion/deletion or splice-site variants (Kluska et al., 2019; Shanmugavel et al., 2019). Haplotype studies have shown a founder effect for this prevalent pathogenic variant, which results in the conversion of arginine to leucine, a significant reduction in the level of copper-transporting ATPase, and enhanced degradation (Bartee and Lutsenko, 2007). Some studies have shown a correlation between the R778L mutation in the *ATP7B* gene and clinical indicators, such as age at onset, clinical presentation, and serum copper-cyanophorin (CP) concentration (Xue et al., 2023; Gu et al., 2005). This mutation has been shown to be associated with a significant reduction in the level of copper-transporting ATPases and an increase in degradation capacity (Zhang et al., 2022). The limited number of patients in each study may have reduced the ability to analyze genotype-phenotype correlations, leading to discrepancies.

Therefore, the primary aim of this study was to evaluate age-stratified clinical and biochemical characteristics at disease onset, while secondarily exploring the genetic landscape and genotype–phenotype associations in this cohort. We collected and analyzed data from children living in Southwest China. Considering the need for early, noninvasive identification of steatosis in pediatric patients with WD, routine clinical and biochemical correlations were examined to support actual screening and to explore their codes in children with WD.

## Methods

### Patients and data collection

One hundred and seventy patients with WD were recruited from January 2018 to October 2025 at Children’s Hospital of Chongqing Medical University, with an age range of pediatric patients was from 0 to 18 years old. Patients were recruited from a single tertiary referral center in Southwest China. Their registered residences covered multiple provinces, predominantly Chongqing (n = 59), Yunnan (n = 37), Sichuan (n = 35), and Guizhou (n = 24), with additional cases from Hubei, Hunan, Shaanxi, and Guangdong. Medical history, physical examination results, laboratory test results, imaging results and liver puncture results were collected as clinical data. Physical examination included examination of jaundice, hepatomegaly, Kayser-Fleischer (K-F) rings, and various types of neurological symptoms. Laboratory tests included routine blood tests, lipid tests, liver biochemistry, coagulation tests, virological tests (hepatitis viruses, cytomegalovirus, and Epstein–Barr virus), ceruloplasmin, and 24-h urinary copper levels and/or random urinary copper. The imaging tests included liver ultrasonography, liver computed tomography (CT) and brain MRI. All clinical and biochemical data were collected at the time of disease onset, prior to any treatment, to avoid potential confounding effects of therapy, and diagnoses were made based on patients’ clinical symptoms, biochemical parameters, and/or genetic analysis. All children were evaluated using the Leipzig score, with a total score of ≥4 in each case. The children were divided into four age-segregated groups, the first being 0–4, the second 5–8, the third 9–12, and the fourth 13–18 years. Eighty-one children underwent direct sequencing of the *ATP7B* gene, and variant analysis was performed using a standard protocol for extracting genomic DNA from children’s peripheral blood leukocytes. All 21 exons were sequenced. Written informed consent was obtained from the guardians of all participants. This study was conducted in accordance with the Declaration of Helsinki and was approved by the Ethics Committee of Children’s Hospital of the Chongqing Medical University. File No. 2025 (190).

### Phenotype definition and genotype-phenotype evaluation

Genetic testing was performed in patients for whom sequencing was clinically indicated (e.g., diagnostic uncertainty, atypical presentation, or family counseling needs) and for whom written informed consent was obtained from their guardians. Patients without genetic data were diagnosed based on clinical and biochemical criteria alone (Leipzig score ≥ 4). Baseline demographic and clinical characteristics were compared between the sequenced and non-sequenced patients, and no statistically significant differences were observed in age at onset, sex distribution, or predominant clinical phenotype (hepatic vs. neurological), suggesting that the sequenced subgroup was broadly representative of the overall cohort. Age at onset and presenting symptoms were used as defining markers of the WD phenotypes. Patients presenting with clinically active hepatic manifestations—including jaundice, anorexia, nausea, coagulopathy, and ascites—were classified according to hepatic subtypes. Patients with predominant neurological features, such as dystonia, tremor, gait disturbance, or dysphagia, with or without concomitant hepatic involvement, or with brain MRI findings indicative of neurological lesions, were classified into the neurological subtype.

Patients identified incidentally during physical examinations were categorized as asymptomatic because they may subsequently develop symptomatic hepatic or neurological diseases. A small subset of patients presenting with severe manifestations involving other organ systems at disease onset, such as arthralgia or arthritis, hematologic abnormalities, or renal symptoms, was classified as the mixed subtype. Given that the majority of patients in this study were pediatric and that hepatic injury was predominantly observed in the early stages, with a particular focus on hepatic steatosis, patients with significant hepatic involvement were further stratified into two groups: hepatic steatosis and hepatic fibrosis. Hepatic steatosis was defined based on the histopathological findings, including hepatocellular steatosis with ballooning degeneration, occasional vacuolated nuclei, and intracellular lipid droplets observed under a microscope. Hepatic fibrosis was defined by imaging findings suggestive of fibrosis on abdominal ultrasound or CT, or by liver stiffness measurement ≥5 kPa assessed using shear wave elastography. Genotype–phenotype correlation analyses were subsequently performed in patients with hepatic or neurological involvement.

### Statistical analysis

All statistical analyses were performed using SPSS (version 27.0; IBM Corp., Armonk, NY, USA) and R software (version 4.4.1; R Foundation for Statistical Computing, Vienna, Austria). Continuous variables were expressed as mean ± standard deviation (SD) or median with interquartile range (IQR), depending on the data distribution, which was assessed using the Shapiro–Wilk test. Categorical variables were summarized as frequencies and percentages.

Comparisons among multiple groups were performed using one-way analysis of variance (ANOVA) for normally distributed variables or the Kruskal–Wallis test for non-normally distributed variables, followed by *post hoc* pairwise comparisons with Bonferroni or Dunn’s correction, as appropriate. For comparisons between two groups, the independent samples t-test or Mann–Whitney U test was used. The chi-squared test or Fisher’s exact test was used to compare categorical variables. Univariate and multivariate logistic regression analyses were performed to evaluate the association between *ATP7B* variants and clinical outcomes. Variables with potential clinical relevance (p < 0.10 in the univariate analysis were included in the multivariable models. The results were reported as odds ratios (ORs) with 95% confidence intervals (CIs). Correlation analyses were performed using Spearman’s rank correlation coefficients. A two-sided p-value < 0.05 was considered statistically significant. For the genetic analysis, variant frequency distributions were calculated across exons and introns. Co-occurrence network analysis of *ATP7B* variants was performed using R (igraph package), where nodes represent individual variants and edges represent co-occurrence within the same patient. The node size is proportional to the variant frequency, and the edge weight reflects the co-occurrence frequency. The rear edges were filtered for the clarity of visualization. Multiple comparisons were adjusted using the (Bonferroni/FDR) method where appropriate. All statistical tests were two-tailed, and the significance thresholds were adjusted for multiple comparisons, where applicable.

## Results

### Relationship between gender, age of onset and clinical phenotype of WD

In total, 170 pediatric patients meeting the predefined inclusion criteria were enrolled, comprising 101 boys (59.17%, mean age 6.31 ± 3.45 years) and 69 girls (40.83%, mean age 6.87 ± 3.40 years), with male children are more likely to be affected and a relatively uniform age distribution across the cohort. With respect to clinical presentation at the time of diagnosis, hepatic involvement was the predominant manifestation (n = 143, 84.12%, mean age 5.89 ± 2.92 years), followed by mixed hepatic/neurological/multisystem involvement (n = 19, 11.18%, mean age 9.81 ± 3.50 years), isolated neurological presentation (n = 6, 3.53%, mean age 12.60 ± 2.89 years), and asymptomatic cases (n = 2, 1.17%, mean age 3.50 ± 3.54 years). Notably, patients presenting with neurological or mixed-type involvement were significantly older than those with isolated hepatic manifestations, suggesting age-dependent phenotypic progression. K-F ring positivity was identified in 50 patients (29.4%), and this subgroup exhibited a significantly higher mean age at diagnosis compared to K-F ring-negative patients (9.39 ± 3.10 vs. 5.35 ± 2.81 years, p < 0.01). Hepatic steatosis and/or hepatic fibrosis was documented in 66 patients (38.8%), who were likewise significantly older at diagnosis than patients without hepatic steatosis and/or fibrosis (8.07 ± 3.64 vs. 5.56 ± 2.91 years, p < 0.01). Abnormal cranial MRI findings were detected in 53 patients (31.2%), and this subgroup demonstrated a significantly greater mean age at diagnosis than patients with normal neuroimaging findings (8.56 ± 4.16 vs. 5.62 ± 2.58 years, p < 0.01). Collectively, these data indicated a significant positive correlation between age at disease onset and the severity of clinical and radiological manifestations, including K-F ring positivity, hepatic steatosis, cirrhosis, and abnormal cranial MRI findings, suggesting that advancing age at presentation is associated with more advanced and multisystem involvement in pediatric WD (Table 1).

**TABLE 1:** Age at onset and clinical characteristics of the enrolled pediatric patients with Wilson’s Disease.

Parameters	Number	Age	%
Gender			
Male	101	6.31 ± 3.45	59.17%
Female	69	6.87 ± 3.40	40.83%
Clinical presentation			
Hepatic	143	5.89 ± 2.92	84.12%
Neurologic	6	12.60 ± 2.89	3.53%
Mixed	19	9.81 ± 3.50	11.18%
Renal abnormalities	6	11.67 ± 4.18	3.53%
Hematologic alterations	9	8.57 ± 2.23	5.29%
Musculoskeletal involvement	4	9.81 ± 4.47	2.36%
Asymptomatic	2	3.5 ± 3.54	1.17%
Kayser-fleischer ring			
Positive	50	9.39 ± 3.10**	29.4%
Negative	120	5.35 ± 2.81	70.6%
Hepatic steatosis-cirrhosis			
Positive	66	8.07 ± 3.64**	38.8%
Negative	104	5.56 ± 2.91	61.2%
Abnormal cranial MRI			
Positive	53	8.56 ± 4.16**	31.2%
Negative	117	5.62 ± 2.58	68.8%

** p < 0.001.

**FIGURE 1:**
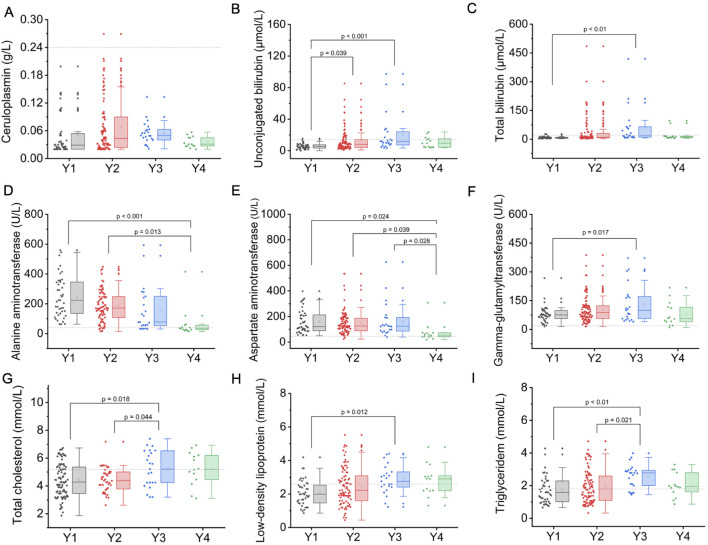
Hepatic biochemical parameters and lipid profiles stratified by age at disease onset in pediatric patients with Wilson’s disease. Scatterplots and stacked box plots illustrating the distribution of liver and lipids in four age groups of onset: Y1 (0–4 years), Y2 (5–8 years), Y3 (9–12 years) and Y4 (13–18 years). **(A)** Distribution of ceruloplasmin levels (g/L) across age-at-onset groups; the horizontal dashed line indicates the lower limit of the normal reference range (0.24 g/L). **(B)** Distribution of unconjugated bilirubin concentrations (µmol/L) across age-at-onset groups. **(C)** Distribution of total bilirubin concentrations (µmol/L) across age-at-onset groups. **(D)** Distribution of ALT activity (U/L) across age-at-onset groups. **(E)** Distribution of AST activity (U/L) across age-at-onset groups. **(F)** Distribution of GGT activity (U/L) across age-at-onset groups. **(G)** Distribution of total cholesterol concentrations (mmol/L) across age-at-onset groups. **(H)** Distribution of LDL cholesterol concentrations (mmol/L) across age-at-onset groups. **(I)** Distribution of triglyceride concentrations (mmol/L) across age-at-onset groups. (Data are presented as scatter plots with superimposed box plots. Horizontal brackets with p-values indicate statistically significant between-group differences determined by the Kruskal–Wallis test followed by Dunn’s *post hoc* correction for multiple comparisons. Statistical significance was set at p < 0.05).

Given the significant variation in age at symptom onset identified in the initial analysis, the patients were stratified into four age-based groups to identify clinically meaningful factors relevant to early diagnosis: Y1 (0–4 years), Y2 (5–8 years), Y3 (9–12 years), and Y4 (13–18 years). The distribution of patients across age groups was as follows: Y1 (n = 43), Y2 (n = 90), Y3 (n = 23), and Y4 (n = 14). Comparative analysis of ceruloplasmin levels, hepatic biochemical parameters, and lipid profiles across the four groups revealed several statistically significant between-group differences. Ceruloplasmin levels were reduced in all patients, regardless of their age at onset (Figure 1A), with no statistically significant differences observed among the four groups. Unconjugated bilirubin concentrations increased stepwise from Y1 to Y3 (Figure 1B), with the first statistically significant increase occurring at Y2 relative to Y1 (p = 0.039). This upward trend persisted in Y3, where unconjugated bilirubin levels were significantly higher than those in Y1 (p < 0.001), and was accompanied by a progressively wider interquartile range and increased outlier dispersion. Total bilirubin levels followed a similar trajectory (Figure 1C), demonstrating a clear and progressive increase across the prepubertal age range; levels in Y3 (9–12 years) were significantly higher than those in Y1 (0–4 years) (p < 0.01). Notably, the Y3 group exhibited substantially greater data dispersion, with individual values approaching or exceeding 300–450 µmol/L, suggesting that a subset of children presenting in the 9–12 years age window may already be in an acute phase of hepatic injury at the time of diagnosis. Alanine aminotransferase (ALT), a sensitive marker of hepatocellular integrity, was elevated above the normal range in all age groups (Figure 1D). ALT levels were generally highest in children before the age of 8 years, with a tendency toward lower, and in some cases normal, values in those with disease onset at or after 9 years of age. ALT levels were significantly higher in Y1 than in Y3 (p = 0.013) or Y4 (p < 0.001). Aspartate aminotransferase (AST) demonstrated a parallel trajectory (Figure 1E), with significant pairwise differences among Y1 and Y3 (p = 0.024), Y1 and Y4 (p = 0.039), and Y3 and Y4 (p = 0.028). Gamma-glutamyltransferase (GGT), a marker of hepatocellular injury and biliary tract involvement, was significantly elevated in Y1 compared with Y4 (p = 0.017) (Figure 1F). With respect to lipid metabolism, total cholesterol was significantly elevated in children with disease onset in Y3 compared to Y1 (p = 0.018) and Y4 compared with Y2 (p = 0.044), with above-normal total cholesterol values increasingly prevalent in children presenting after the age of 9 years (Figure 1G). Low-density lipoprotein (LDL) levels gradually increased above the upper limit of normal from approximately 5 years of age onward, with a statistically significant difference between Y3 and Y1 (p = 0.012) (Figure 1H). Triglyceride levels were significantly higher in Y1 than in Y3 (p < 0.01) and a significant difference was observed between Y2 and Y3 (p = 0.021) (Figure 1I), indicating a dynamic and age-dependent pattern of lipid metabolic dysregulation across the disease course.

**FIGURE 2:**
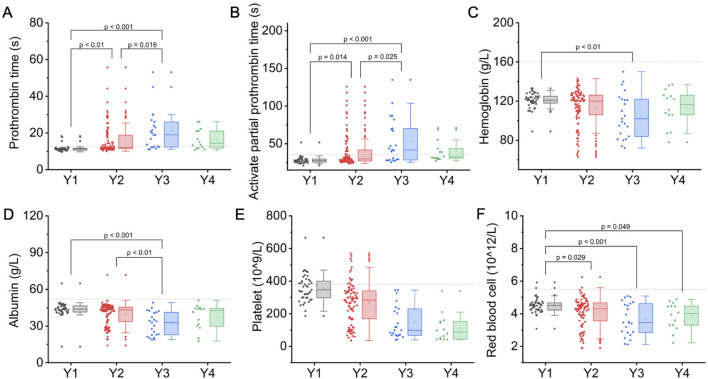
Age of onset and coagulation and hematologic indices in pediatric patients with Wilson’s disease. **(A)** Distribution of prothrombin time (PT) by age of onset group. **(B)** Distribution of activated partial thromboplastin time (APTT) by age of onset group. **(C)** Distribution of hemoglobin concentration by age of onset group. **(D)** Distribution of serum albumin concentration by age of onset group. **(E)** Distribution of platelet count (×10^9^/L) in the onset age group. **(F)** Distribution of red blood cell counts in the age group of onset (×10^12^/L.). Data are presented as scatter plots with superimposed box plots (Horizontal brackets with p-values indicate statistically significant between-group differences determined by the Kruskal–Wallis test followed by Dunn’s *post hoc* correction for multiple comparisons. Statistical significance was set at p < 0.05).

To assess the relationship between laboratory parameters and age of onset in patients with WD, we divided the patients into four age groups for comparison. Compared with the youngest age-at-onset cohort (Y1), prothrombin time (PT) (Figure 2A) and activated partial thromboplastin time (APTT) (Figure 2B) were significantly prolonged in group Y2 (PT, p < 0.01; APTT, p = 0.014) and remained significantly elevated in group Y3 compared to group Y1 (PT and APTT, p < 0.001). In addition, PT and APTT were significantly higher in Y3 than in Y2 (PT, p = 0.016; APTT, p = 0.025), suggesting that coagulation function progressively deteriorates with the age of onset. Serum albumin levels decreased significantly from Y1 to Y3 (p < 0.001) and from Y1 to Y4 (p < 0.01) but remained at normal levels in most children, with some elevation in children with a high age of onset. Hemoglobin levels (Figure 2C), albumin (Figure 2D), platelet counts (Figure 2E), and red blood cell (Figure 2F) levels varied significantly among the four age groups, mainly because of the wide individual variation, and remained largely below the upper limit of normal levels, with no deviation from normal values in the larger data group.

### Correlation between genotype and phenotype

**FIGURE 3:**
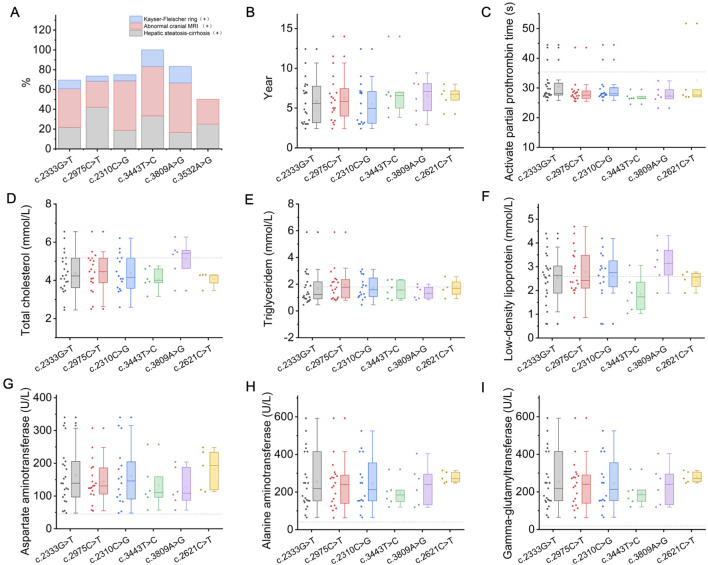
Relationship between phenotype and clinical manifestations of the six high-frequency *ATP7B* mutation loci. **(A)** Distribution of Kayser-Fleischer ring positivity, cranial MRI abnormalities, and hepatic steatosis/fibrosis among children carrying each of the six high-frequency mutations. **(B)** Age distribution of children across the six mutation groups. **(C)** Activated partial thromboplastin time in children stratified by mutation. **(D–F)** Serum total cholesterol, triglyceride, and low-density lipoprotein levels across mutation groups. **(G–I)** Serum aspartate aminotransferase, ALT, and gamma-glutamyltransferase levels across mutation groups.

Six high-frequency *ATP7B* nucleotide variants were identified in this cohort of children from Southwest China (Figure 3A): c.2333G>T (p.Arg778Leu), c.2975C>T (p.Pro992Leu), c.2310C>G (p.Leu770 =), c.3443T>C (p.Ile1148Thr), c.3809A>G (p.Asn1270Ser), and c.2621C>T (p.Ala874Val). K-F rings were detected in relatively high proportions across most variant groups, with notably higher frequencies observed in the c.2975C>T and c.2310C>G groups. Abnormal cranial MRI findings were particularly prevalent in patients carrying the c.2333G>T and c.2975C>T variants. Hepatic steatosis or cirrhosis was present across all mutation groups at broadly similar proportions, with the highest frequency observed in c.3532A>G (p.Thr1178Ala) carriers. With respect to age at the time of mutation detection (Figure 3B), median age was broadly comparable across most groups, ranging from approximately 5 to 8 years, although substantial within-group variability was noted. APTT values showed a similar distribution across the six variants, with the majority of patients falling between 25 s and 35 s and a small number of outliers exceeding 40 s (Figure 3C). Regarding blood lipids, total cholesterol levels were broadly similar across the variant groups, with median values approximating the reference threshold indicated by the dashed line (Figure 3D). Triglyceride levels were similar among the groups, with most values below 3 mmol/L, although outliers were identified in the c.2975C>T and c.2310C>G groups (Figure 3E). LDL concentrations showed overlapping distributions across all six variants (Figure 3F). Regarding liver biochemistry (Figures 3G–I), AST, ALT, and GGT demonstrated marked inter-individual variability within each variant group. The c.2333G>T group tended to exhibit higher median enzyme levels and a broader distribution across all three hepatic markers than the other groups, suggesting that this variant may be associated with more severe hepatocellular injury.

### Distribution of *ATP7B* variants and their association with clinical phenotypes

**FIGURE 4:**
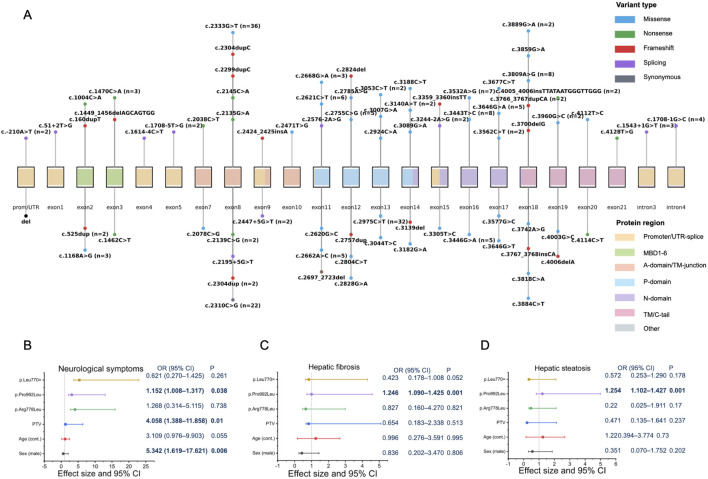
Distribution of *ATP7B* variants and their association with clinical phenotypes. **(A)** Schematic of the distribution of *ATP7B* variants throughout the gene, including promoter/UTR regions, exons and introns. Variants are annotated by type (missense, nonsense, frameshift, splice, and synonymous) and mapped to the corresponding protein regions (promoter/UTR-splice, MBD1-6, A-domain/TM junction, P-domain, N-domain, and TM/C-tail). The number of cases for each variant is shown in parentheses. **(B–D)** Forest plots showing associations between selected *ATP7B* variants and clinical phenotypes including neurological symptoms **(B)**, hepatic fibrosis **(C)**, and hepatic steatosis **(D)**. 95% confidence intervals (CIs) for the ratio of ratios (ORs) (Statistical significance was determined by logistic regression analysis with corresponding p values).

**FIGURE 5:**
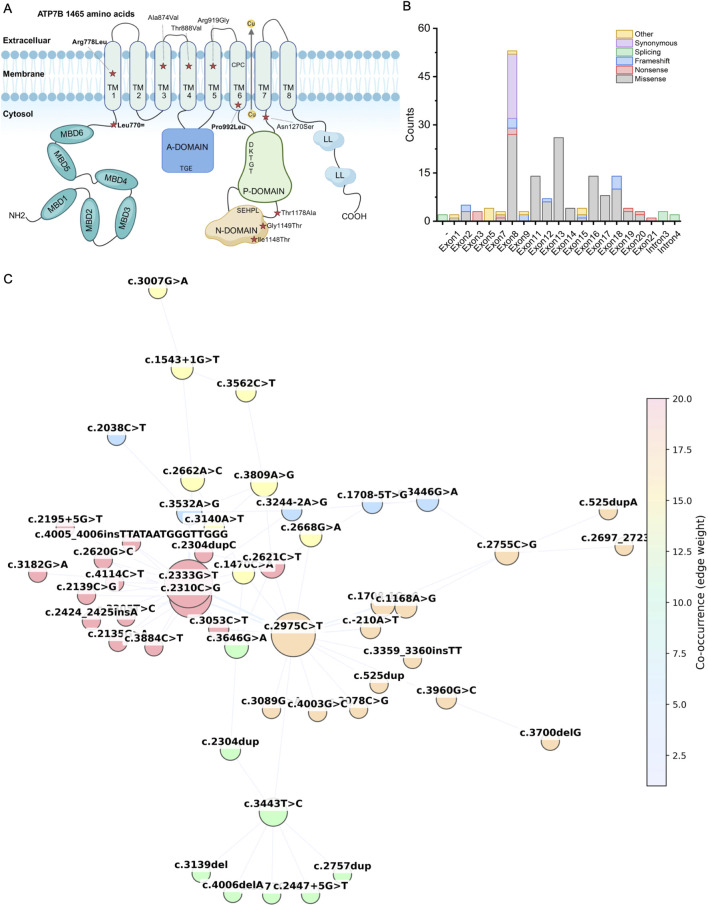
Structural mapping, exon distribution, and co-occurrence network of *ATP7B* variants in pediatric WD. **(A)** Schematic representation of the ATP7B protein structure, showing the localization of the top ten most frequent variants across functional domains. **(B)** Distribution of *ATP7B* variants across exonic and intronic regions. **(C)** Co-occurrence network of *ATP7B* variants. Each node represents a variant, with node size proportional to its frequency (Edges indicate the co-occurrence of variants within the same individual, and the thickness and color intensity of the edges correspond to the co-occurrence weight).

There were 76 distinct *ATP7B* variants identified across multiple functional domains (Figures 4A, 5A), including the promoter/UTR-splice region, metal-binding domains (MBD1–6), A-domain/TM linkage, P-domain, N-domain, and TM/C-tail region. These variants were broadly distributed across the promoter/UTR, exonic, and intronic regions, with no evidence of a single predominant hotspot. Missense mutations were the most frequent, followed by frameshift and splice-site variants, whereas nonsense and synonymous variants were relatively uncommon. Among these, c.2333G>T (n = 36), c.2310C>G (n = 22), and c.2975C>T (n = 32) were the most prevalent allelic alterations in the pediatric cohort. Logistic regression analyses were conducted to identify independent predictors of primary clinical outcomes. In the analysis of neurological symptoms (Figure 4B), the p.Pro992Leu variant was significantly associated with an increased risk (OR = 1.152, 95% CI: 1.008–1.317, p = 0.038). Protein-truncating variants (PTVs) were also significantly associated with a higher risk of neurological symptoms (OR = 4.058, 95% CI: 1.388–11.858, p = 0.01), and male sex was identified as an independent risk factor (OR = 5.342, 95% CI: 1.619–17.621, p = 0.006). No statistically significant associations were observed among the remaining variants. In contrast, no significant association was observed between the analyzed variants and hepatic fibrosis, although a tendency to reduced risk was noted for p.Leu770Leu (OR = 0.423, 95% CI: 0.178–1.008, p = 0.052) (Figure 4C). For hepatic steatosis (Figure 4D), the p.Pro992Leu variant was significantly associated with increased risk (OR = 1.254, 95% CI: 1.102–1.427, p = 0.001), whereas no significant associations were observed for other variants or clinical variables, including age and sex. Overall, *ATP7B* variants were widely distributed throughout the gene and may contribute to phenotypic heterogeneity, with specific variants, such as p.Pro992Leu, showing significant associations with certain clinical manifestations.

### Mutational spectrum of *ATP7B* in pediatric patients from southwest China

In children with WD from Southwest China, the top ten high-frequency mutation loci were mapped to the ATP7B protein structure (Figure 5A). The frequency statistics of variants by the number of occurrences of all alleles present in the children in the cohort can be viewed in conjunction with the protein structure map (Supplementary Table S1). Comprehensive mutational profiling of *ATP7B* in this cohort revealed a diverse and exon-skewed distribution of pathogenic variants. As shown in the superimposed bar plots (Figure 5B), exon 8 exhibited the highest mutation burden (approximately 49 allele counts) and represented the predominant mutational hotspot, with missense variants accounting for the majority of alleles, alongside frameshift, nonsense, and synonymous variants. Exons 13 and 12 harbored the second and third highest mutation frequencies, with approximately 27 and 14 mutations, respectively, predominantly consisting of missense variants. Exon 18 showed a relatively higher proportion of frameshift mutations than other exons. Other exons, including 9, 15, 16, and 17, exhibited moderate mutation frequencies, whereas the remaining exons and intronic regions (introns 3 and 4) exhibited relatively few variants. Across all exons, missense mutations were the most prevalent variant type, followed by frameshift, splice-site, nonsense, synonymous, and other variants. Co-occurrence network analysis revealed a compound heterozygous architecture in the cohort (Figure 5C). Notably, c.2975C>T emerged as the most central and highly connected node, displaying the greatest number of co-occurring interactions and the highest edge weight, indicating frequent co-occurrence with secondary alleles, such as c.2333G>T, c.2310C>G, c.3646G>A, and c.3053C>T. In contrast, several variants, including c.3443T>C, c.2304dup, and c.3139del, formed relatively isolated clusters with limited cross-allelic interactions, suggesting that they predominantly occur in homozygous or rare compound heterozygous configurations. Collectively, these findings indicate that exon 8 is the principal mutational hotspot of *ATP7B* in pediatric patients with WD from Southwest China and highlight c.2975C>T as the most common compound heterozygous allele in this cohort, providing important insights into the regional mutational architecture of the disease.

### Combinatorial profiles of *ATP7B* mutations in genotyped pediatric patients

**FIGURE 6:**
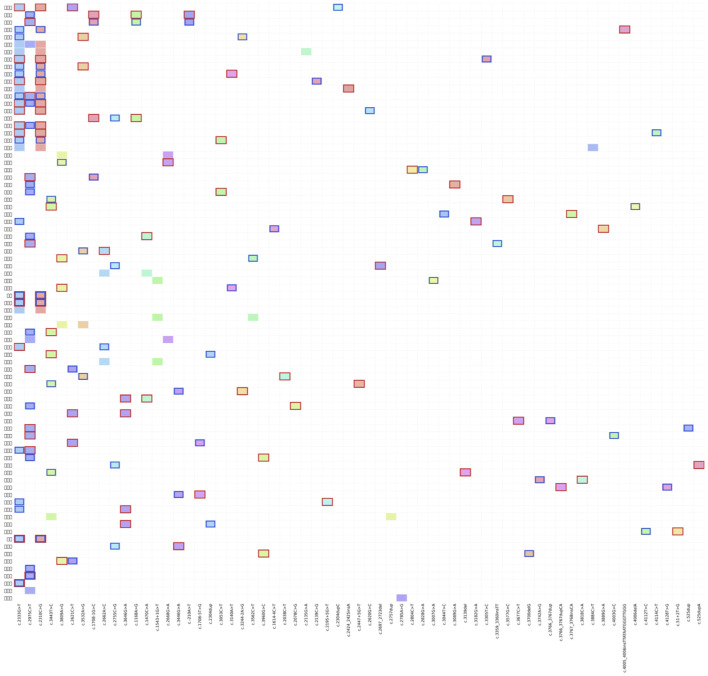
Distribution of *ATP7B* variants in children with pediatric Wilson’s disease. Each row represents each child and each column represents a specific *ATP7B* variant, with different colors corresponding to different variant types. Variant clusters are recurrent high-frequency combinations of variants. Blue outer boxes indicate variants carried and inherited by the father, and red outer boxes indicate variants carried and inherited by the mother.

The analysis of compound heterozygous *ATP7B* mutation combinations (Supplementary Table S2) in pediatric patients with WD (Figure 6) revealed a complex and highly heterogeneous pattern of variant pairing. Unique or low-frequency allele combinations were observed in most patients, reflecting the extensive allelic heterogeneity of the Southwest China cohort. Among the identified compound heterozygous pairs, combinations involving c.2333G>T, c.2975C>T, and c.2310C>G as constituent alleles were most frequently observed across multiple rows and columns, consistent with their status as high-frequency variants within the cohort. Notably, the c.2333G>T/c.2975C>T combination represented one of the most frequent allele pairings and formed a prominent cluster in the upper-left region of the matrix, indicating recurrent co-occurrence in multiple patients. Individual patients exhibited a wide range of variant-type combinations, including missense, frameshift, missense/nonsense, and missense/splice-site pairs. However, no single combination was predominant across the cohort. Rare variants, including large insertions (e.g., c.4005_4006insTTATAATGGGGTTGGG) and structural duplications, were observed only in isolated pairs with common alleles. Parental origin analysis demonstrated that the variants outlined in blue were inherited from the father, whereas those outlined in red were inherited from the mother (Figure 6). In most cases, the two alleles identified in the affected child were derived from different parental carriers, supporting the classical autosomal recessive inheritance pattern of WD. Furthermore, recurrent variants such as c.2333G>T, c.2975C>T, and c.2310C>G were frequently observed in combination with multiple secondary alleles, suggesting a central role in shaping the mutational architecture of the cohort. In contrast, a subset of variants appeared as isolated events with limited co-occurrence, indicating that they may occur in heterozygous states or rare compound heterozygous configurations. The overall sparse and widely distributed pattern of data points within the matrix underscores the substantial diversity of compound heterozygous combinations, with most allelic pairings identified in only one or two affected individuals.

## Discussion

This study systematically characterized the clinical, biochemical, and genetic features of Wilson disease (WD) in a cohort from Southwest China. We observed clear age-related differences in clinical presentation, with hepatic manifestations predominating in patients with younger ages of onset patients and neurological involvement emerging at older ages of onset. Biochemical analyses suggested a shift from hepatocellular injury to metabolic dysfunction with increasing age of onset. In addition, genotype–phenotype analysis revealed variant-associated clinical tendencies among common *ATP7B* mutations, while the mutational spectrum expanded to include rare exonic and intronic variants, reflecting the genetic complexity of pediatric WD.

The clinical characteristics of this cohort were consistent with those of previous pediatric WD studies. Most patients presented with hepatic involvement, whereas neurological and mixed phenotypes were more frequent in older children, supporting the progression from hepatic copper accumulation to systemic and neurological involvement (Gu et al., 2005; Dong et al., 2024). Older age at onset was also associated with higher frequencies of K-Frings, hepatic steatosis/cirrhosis, and abnormal brain MRI findings, highlighting the progressive nature of copper deposition and the importance of early screening to prevent organ damage. Age-stratified biochemical analyses suggest that disease progression is accompanied by metabolic remodeling. ALT, AST, and GGT levels gradually decline with increasing age at onset, whereas total cholesterol and LDL levels increase, indicating worsening lipid dysregulation despite reduced levels of liver injury markers (Berenguer et al., 2025). PT and APTT were progressively prolonged, whereas serum albumin levels declined, indicating impaired hepatic synthetic function due to cumulative Cu toxicity (Schaefer et al., 2015; Auth et al., 2005). Platelet counts show only modest changes, suggesting that thrombocytopenia at an older age of onset is more likely related to hypersplenism rather than direct copper toxicity (Zhong et al., 2020; Wang et al., 2022). In addition, discrepancies between red blood cell counts and hemoglobin levels may indicate subclinical hemolysis, consistent with the known role of copper-induced oxidative damage in erythrocytes (Segel and Lichtman, 2014). These findings indicate that late onset is associated with more advanced hepatic dysfunction and hematological abnormalities.

Insights into disease progression can be partially explained by genotype–phenotype correlations. The present study confirms and extends previous observations regarding the relationship between *ATP7B* variants and clinical phenotypes of pediatric WD in China. Among the six high-frequency variants analyzed—c.2333G>T, c.2975C>T, c.2310C>G, c.3443T>C, c.3809A>G, and c.2621C>T—substantial overlap was observed in the distribution of clinical manifestations, including K-F ring positivity, hepatic steatosis/cirrhosis, and abnormal brain MRI findings across groups. This is consistent with the widely recognized difficulty in establishing robust genotype–phenotype correlations in WD. However, some variant-specific trends were also observed. Higher proportions of K-F ring positivity and abnormal brain MRI findings were noted in the c.2975C>T and c.2310C>G groups, which is consistent with previous reports indicating that c.2975C>T was significantly associated with earlier disease onset (p = 0.002), which in turn correlated with greater cumulative copper deposition and more advanced clinical phenotypes at diagnosis. Similarly, patients harboring c.2975C>T or c.3809A>G have been reported to develop WD manifestations predominantly before the age of 12 years, a pattern reflected by the lower median age observed in these groups in our cohort (Vrabelova et al., 2005). In contrast, the c.3443T>C group exhibited a relatively older age distribution, which is in line with prior findings that carriers of this variant tend to present after 12 years of age. This observation suggests that c.3443T>C may confer partially preserved *ATP7B* function and slow copper accumulation kinetics (Dong et al., 2016). This interpretation is supported by the molecular localization of Ile1148 within the transmembrane domain of ATP7B, where partial disruption of channel function rather than complete loss may delay phenotypic expression. With respect to hepatic biochemistry, patients in the c.2333G>T group demonstrated the widest distribution and highest median levels of AST, ALT, and GGT among all the variant groups, corroborating previous evidence that the Arg778Leu mutation exerts a severe functional effect on ATP7B and is associated with pronounced hepatocellular injury. Moreover, c.2333G>T homozygotes have been reported to exhibit significantly lower ceruloplasmin levels and earlier hepatic manifestations than compound heterozygotes (Wu et al., 2001), suggesting that the severity of biochemical liver injury is at least partially dependent on allele dosage. In our study, patients carrying homozygous c.2333G>T, often in linkage to c.2310C>G, tended to exhibit more severe phenotypes than heterozygous carriers. However, owing to the limited number of patients with homozygous mutations for variants other than c.2333G>T, systematic comparisons between the homozygous and compound heterozygous states were not feasible. Therefore, no definitive conclusions can be drawn regarding the potential genotype dosage effects, which require further investigation in larger cohorts. The relatively high prevalence of hepatic steatosis and cirrhosis observed in the c.3532A>G mutation group is noteworthy. Although c.3532A>G has been primarily reported in hepatic phenotype-dominant patients in southern Chinese cohorts (Peña-Quintana et al., 2012; Li et al., 2021), its association with a high burden of structural liver disease in pediatric populations has not been well characterized in prior genotype–phenotype analyses. This may reflect the regional enrichment of specific variants in Southwest China. Because ATP7B sequencing was only available for a subset of patients, a potential selection bias could not be excluded, particularly in the genotype-phenotype correlation analyses. Therefore, the results should be interpreted with caution.

Coagulation parameters, including APTT, exhibited broadly similar distributions across all six variant groups, indicating that coagulation abnormalities in WD are primarily driven by the degree of hepatic synthetic dysfunction, rather than by specific mutation types. Likewise, lipid parameters, including total cholesterol, triglycerides, and LDL, showed largely overlapping interquartile ranges across the groups, suggesting the absence of variant-specific effects on hepatic lipid metabolism. These findings align with the broader consensus that biochemical severity at any given time point more accurately reflects disease stage and treatment exposure than the genotype alone. In a landmark Chinese cohort of 1,302 patients with WD, multivariable logistic regression identified older age at onset, rather than mutation type, sex, or protein-truncating variant status, as the primary factor associated with neurological manifestations, underscoring that WD phenotypes are fundamentally shaped by the temporal dynamics of copper accumulation rather than isolated genetic determinants (Zhang et al., 2022). Notably, c.2621C>T (p.Ala874Val) appeared disproportionately in the mixed neurological and hepatic phenotype group in the present study. It has been hypothesized that concurrent substitution of valine at position 874 with leucine at position 778 synergistically destabilizes the transmembrane domain structure of ATP7B. In this context, the co-occurrence of c.2621C>T and c.2333G>T observed in several patients in our cohort may represent a potentially deleterious combinatorial effect, which warrants further investigation.

Genotype–phenotype analysis in this cohort further highlights the complexity of clinical expression in pediatric WD. Consistent with previous studies, a substantial overlap in clinical manifestations was observed across common *ATP7B* variants, indicating that robust genotype–phenotype correlations remain difficult to establish. However, several variant-associated trends were observed. Overall, the genotype–phenotype data presented here reinforce the concept that although certain ATP7B variants are associated with specific clinical tendencies, such as earlier onset for c.2975C>T and c.3809A>G, more severe hepatocellular injury for c.2333G>T, and later onset for c.3443T>C, no single variant can reliably predict the clinical outcomes in individual pediatric patients. Therefore, genotyping should be interpreted in conjunction with comprehensive clinical and biochemical assessments, rather than used in isolation for prognostic purposes.

The mutational landscape identified in this cohort both confirms and meaningfully expands the established spectrum of ATP7B variants in Chinese pediatric patients with WD. c.2333G>T and c.2975C>T consistently represent the highest mutation burden, with allele frequencies of approximately 28.6% and 13.8% (Zhang et al., 2022), respectively. In our dataset, the co-occurrence of c.2333G>T and c.2310C>G as part of a tightly linked haplotype was also observed, with c.2310C>G almost exclusively present in the same allele as c.2333G>T. This is consistent with previous reports indicating that c.2310C>G functions as a synonymous polymorphism in strong linkage disequilibrium with the pathogenic c.2333G>T variant. In addition to these recurrent variants, several less common or potentially novel variants identified in this cohort warrant further investigation. The large insertion c.4005_4006insTTATAATGGGTTGGG (p.Ile1336delinsLeuTer) was detected in a triallelic configuration and was classified as a likely pathogenic in-frame insertion. Its occurrence within a complex multiallelic background is uncommon, and its contribution to phenotypic severity in compound heterozygous states remains unclear. Similarly, the nonsense variant c.2139C>G, which introduces a premature termination codon within the critical A-domain/transmembrane linker region of ATP7B, despite nonsense variants accounting for approximately 9% of all reported pathogenic ATP7B alleles globally (Couchonnal et al., 2021; Koboldt et al., 2020). The c.3532A>G variant, which affects the ATP7B N-domain, was identified in multiple patients in our cohort. Although previously reported predominantly in hepatic phenotype–dominant patients from southern China (Zhang et al., 2022; Li et al., 2021; Wei et al., 2014), its allele frequency remains substantially lower than that of classical hotspot mutations and its inclusion in routine targeted screening panels has not yet been standardized. Among the structural variants, the frameshift mutation c.2304dupC (p.Met769HisfsTer26), which results in the premature truncation of exon 8, is of particular interest. This variant has been previously identified in Chinese patients with WD and classified as pathogenic, although its allele frequency remains relatively low (approximately 2.3% in Chinese cohorts) (Lao and Le, 2024). In our cohort, this variant was observed in both the biallelic and compound heterozygous configurations. In addition, splice-site variants, including c.1708-1G>C, c.1614-4C>T, c.2447+5G>T, c.3244-2A>G, and c.2195+5G>T, represent a class of pathogenic alterations that disrupt canonical splice sites and nearby regulatory elements. Splice-site variants account for approximately 10.5% of the pathogenic ATP7B alleles in Chinese patients, and several variants identified in this study have previously been reported only in isolated cases or single-center series, highlighting the importance of comprehensive intronic sequencing in WD genetic testing (Członkowska et al., 2018). Among the rare compound heterozygous configurations observed in this cohort, several combinations merited particular attention. The co-occurrence of c.1708-1G>C with c.2975C>T and c.1168A>G (p.Ile390Val) in a quadruple-variant case, as well as the combination of c.4005_4006insTTATAATGGGTTGGG with c.2333G>T and c.2310C>G in a triallelic configuration, represent genotypic constellations that, to our knowledge, have not been previously reported.

As suggested by several cases in this cohort, the coexistence of variants affecting both metal-binding domains (MBDs) and transmembrane/executor domains may lead to more profound functional impairment than single-domain mutations alone, although this hypothesis requires further functional validation. Another important finding of this study was the identification of a distinct subset of intronic variants, including canonical splice-site mutations such as c.1543+1G>T and c.1708-1G>C, as well as low-frequency intronic events located within introns 3 and 4. Although less frequent than exonic hotspot mutations, their consistent presence across structural mapping, exon–intron distribution analyses, co-occurrence networks, and patient-level combination matrices indicates that intronic variants constitute a meaningful component of the mutational architecture in pediatric WD. This observation is clinically relevant, as ATP7B intronic defects are increasingly being recognized as an underappreciated source of diagnostic complexity, particularly when conventional exon-focused sequencing fails to fully explain the phenotype. Both c.1543+1G>T and c.1708-1G>C occur at highly conserved splice donor and acceptor sites adjacent to exon boundaries, and are therefore highly likely to disrupt normal pre-mRNA splicing. Recent functional studies have provided direct experimental evidence that these variants alter transcript splicing, supporting their pathogenic classification (Gomes and Dedoussis, 2016). Their recurrent identification in our cohort strongly suggests that they should not be considered incidental findings, but rather *bona fide* disease-associated alleles that may significantly influence the phenotype when paired with common pathogenic variants. Importantly, intronic variants in this cohort were predominantly observed in compound heterozygous configurations with common exonic alleles such as c.2333G>T, c.2975C>T, or c.2310C>G. This pattern is consistent with the autosomal recessive inheritance model of WD and has important diagnostic implications. Reliance solely on hotspot-based exon screening may fail to identify the second pathogenic allele, leading to apparent monoallelic cases and incomplete molecular diagnosis. Increasing evidence indicates that deep intronic ATP7B variants can generate aberrant splice sites or induce exon skipping, while escaping conventional diagnostic pipelines. For example, the recurrent deep intronic variant c.2866-1521G>A has been shown to disrupts ATP7B splicing, whereas c.1947–19T>A induces exon skipping in early onset WD (Liu et al., 2015). Although intronic variants were relatively infrequent, their repeated occurrence—particularly at splice-consensus regions—argues against their classification as benign. Even in the absence of functional validation for every variant, the convergence of positional evidence, co-occurrence with known pathogenic alleles, and consistency with established splicing mechanisms strongly supports their pathogenic potential. Recent transcript-based studies have further demonstrated that synonymous and intronic ATP7B variants may exert pathogenic effects through cryptic splicing defects, emphasizing the need for caution in interpreting noncoding variants of this gene (Gomes and Dedoussis, 2016). Collectively, these findings indicate that intronic ATP7B variants represent a diagnostically and biologically important subset of mutations in pediatric patients with WD. In this cohort, they appeared to function primarily as rare second alleles embedded within a broader compound heterozygous background, rather than as dominant hotspot mutations. This has direct implications for molecular diagnostic strategies: although hotspot-based exon screening remains a useful initial approach in East Asian populations, comprehensive sequencing of exon–intron boundaries—and ideally full-gene or RNA-based analyses in unresolved cases—is necessary to avoid missed diagnoses of splice-disrupting ATP7B defects (Liu et al., 2015).

As this was a retrospective cross-sectional study, the observed differences across age groups reflect age-stratified variation at presentation rather than true longitudinal disease progression. Several limitations of this study should be acknowledged. Although patients were drawn from multiple provinces in Southwest China, this was a single-center study with a relatively limited sample size, which may restrict the generalizability of the findings to broader pediatric WD populations, particularly across different geographic or ethnic backgrounds. Second, although a wide spectrum of *ATP7B* variants was identified, including rare and intronic mutations, functional validation experiments were not performed for all detected variants. Therefore, the pathogenicity of certain low-frequency or novel variants, particularly those with intronic and multiallelic configurations, remains to be clarified. Third, the genotype–phenotype analyses were based primarily on cross-sectional clinical and biochemical data at the time of diagnosis, and longitudinal follow-up data were not systematically incorporated. Future studies incorporating multicenter cohorts, functional assays, transcript-level analyses, and longitudinal clinical follow-up will be essential to further refine genotype–phenotype correlations and clarify the biological significance of rare and noncoding *ATP7B* variants in pediatric WD.

## Data Availability

The original contributions presented in the study are publicly available. This data can be found in the ClinVar repository with the accession numbers from SCV007601324 to SCV007601394.
