# Supplementary material for: Age-associated clinical characteristics and ATP7B mutation landscape in pediatric Wilson’s disease: a study from southwest China
Source: Front Genet. 2026 Jun 16;17:1839993. doi: 10.3389/fgene.2026.1839993 (PMC13313600; doi:10.3389/fgene.2026.1839993)
Supplement: Supplementary file 1 [file Table1.docx]

Table S1.ATP7B nucleotide variants present in the children.

| **Nucleotide change** | **Count** | **Protein change** | **Exon** | **Variant type** | **Splice side** | | **Protein region** | |
| --- | --- | --- | --- | --- | --- | --- | --- | --- |
| **c.2333G>T** | 36(15.6521739130435%) | p.Arg778Leu | 8 | missense | |  | TM-associated / cytosolic loop | |
| **c.2975C>T** | 32(13.9130434782609%) | p.Pro992Leu | 13 | missense | |  | P-domain-enriched | |
| **c.2310C>G** | 22(9.56521739130435%) | p.Leu770= | 8 | synonymous | |  | TM-associated / cytosolic loop | |
| **c.3443T>C** | 8(3.47826086956522%) | p.Ile1148Thr | 16 | missense | |  | N-domain-enriched | |
| **c.3809A>G** | 8(3.47826086956522%) | p.Asn1270Ser | 18 | missense | |  | Core (post-N) | |
| **c.3532A>G** | 7(3.04347826086957%) | p.Thr1178Ala | 16 | missense | |  | N-domain-enriched | |
| **c.2621C>T** | 6(2.60869565217391%) | p.Ala874Val | 11 | missense | |  | P-domain-enriched | |
| **c.2662A>C** | 5(2.17391304347826%) | p.Thr888Pro | 11 | missense | |  | P-domain-enriched | |
| **c.2755C>G** | 5(2.17391304347826%) | p.Arg919Gly | 12 | missense | |  | P-domain-enriched | |
| **c.3446G>A** | 5(2.17391304347826%) | p.Gly1149Glu | 16 | missense | |  | N-domain-enriched | |
| **c.3646G>A** | 5(2.17391304347826%) | p.Val1216Met | 17 | missense | |  | N-domain-enriched | |
| **c.1708-1G>C** | 4(1.73913043478261%) | splicing | intron 4 (splice acceptor of exon 5) | splicing | | acceptor(-) | NA |  |
| **c.1168A>G** | 3(1.30434782608696%) | p.Ile390Val | 2 | missense | |  | N-ter (MBD1–6) | |
| **c.1543+1G>T** | 3(1.30434782608696%) | p.? | intron 3 | splicing | | donor(+) | NA |  |
| **c.-210A>T** | 3(1.30434782608696%) | splicing | 5′ untranslated region (5′ UTR) variant | splicing | |  | NA |  |
| **c.2668G>A** | 3(1.30434782608696%) | p.Val890Met | 11 | missense | |  | P-domain-enriched | |
| **c.1470C>A** | 3(1.30434782608696%) | p.Cys490Ter | 3 | nonsense | |  | N-ter (MBD1–6) | |
| **c.1708-5T>G** | 2(0.869565217391304%) | splicing | 5 | splicing | | acceptor(-) | NA |  |
| **c.2139C>G** | 2(0.869565217391304%) | p.Tyr713Ter | 8 | nonsense | |  | TM-associated / cytosolic loop | |

Continued Table S1.ATP7B nucleotide variants present in the children.

| Nucleotide change | Count | Protein change | Exon | Variant type | Splice side | Protein region | |
| --- | --- | --- | --- | --- | --- | --- | --- |
| **c.2304dup** | 2(0.869565217391304%) | p.Met769HisfsTer26 | 8 | frameshift |  | TM-associated / cytosolic loop | |
| **c.2447+5G>T** | 2(0.869565217391304%) | splicing | 9 | splicing | donor(+) | NA |  |
| **c.525dup** | 2(0.869565217391304%) | p.Val176SerfsTer28 | 2 | frameshift |  | N-ter (MBD1–6) | |
| **c.3053C>T** | 2(0.869565217391304%) | p.Ala1018Val | 13 | missense |  | P-domain-enriched | |
| **c.3140A>T** | 2(0.869565217391304%) | p.Asp1047Val | 14 | missense |  | P-domain-enriched | |
| **c.3244-2A>G** | 2(0.869565217391304%) | splicing | 15 | splicing | acceptor(-) | NA |  |
| **c.3562C>T** | 2(0.869565217391304%) | p.Leu1188Phe | 17 | missense |  | N-domain-enriched | |
| **c.3766_3767dupCA** | 2(0.869565217391304%) | p.Gln1256HisfsTer75 | 18 | frameshift |  | Core (post-N) | |
| **c.3889G>A** | 2(0.869565217391304%) | p.Val1297Ile | 18 | missense |  | Core (post-N) | |
| **c.3960G>C** | 2(0.869565217391304%) | p.Arg1320Ser | 19 | missense |  | Core (post-N) | |
| **c.4005_4006insTTATAATGGGTTGGG** | 2(0.869565217391304%) | p.Gly1335_Ile1336insLeuTer | 19 | nonsense |  | Core (post-N) | |
| **c.1004C>A** | 1(0.434782608695652%) | p.Cys490Ter | 2 | nonsense |  | N-ter (MBD1–6) | |
| **c.1449_1456delAGCAGTGG** | 1(0.434782608695652%) | p.Arg483Serfs*20 | 3 | frameshift |  | N-ter (MBD1–6) | |
| **c.1462C>T** | 1(0.434782608695652%) | p.Gln488Ter | 3 | nonsense |  | N-ter (MBD1–6) | |
| **c.160dupT** | 1(0.434782608695652%) | p.Ser54fs | 2 | frameshift |  | N-ter (MBD1–6) | |
| **c.1614-4C>T** | 1(0.434782608695652%) | splicing | 4 | splicing | acceptor(-) | NA |  |
| **c.2038C>T** | 1(0.434782608695652%) | p.Gln680Ter | 7 | nonsense |  | TM-associated / cytosolic loop | |
| **c.2078C>G** | 1(0.434782608695652%) | p.Ser693Cys | 7 | missense |  | TM-associated / cytosolic loop | |
| **c.2135G>A** | 1(0.434782608695652%) | p.Trp712Ter | 8 | nonsense |  | TM-associated / cytosolic loop | |
| **c.2145C>A** | 1(0.434782608695652%) | p.Tyr715Ter | 8 | nonsense |  | TM-associated / cytosolic loop | |
| **c.2195+5G>T** | 1(0.434782608695652%) | splicing | 8 | splicing | donor(+) | NA |  |
| **c.2299dupC** | 1(0.434782608695652%) | p.Met769Hisfs*2 | 8 | frameshift |  | TM-associated / cytosolic loop | |

Continued Table S1.ATP7B nucleotide variants present in the children.

| Nucleotide change | Count | Protein change | Exon | Variant type | Splice side | Protein region | |
| --- | --- | --- | --- | --- | --- | --- | --- |
| **c.2304dupC** | 1(0.434782608695652%) | p.Met769Hisfs*26 | 8 | frameshift |  | TM-associated / cytosolic loop | |
| **c.2424_2425insA** | 1(0.434782608695652%) | p.Ser693Cys | 9 | frameshift |  | TM-associated / cytosolic loop | |
| **c.2471T>G** | 1(0.434782608695652%) | p.Leu824Arg | 10 | missense |  | TM-associated / cytosolic loop | |
| **c.2576-2A>G** | 1(0.434782608695652%) | splicing | 11 | splicing | acceptor(-) | NA |  |
| **c.2620G>C** | 1(0.434782608695652%) | p.Ala874Pro | 11 | missense |  | P-domain-enriched | |
| **c.2697_2723del** | 1(0.434782608695652%) | p.Ile899_Gln907del | 11 | inframe indel | | P-domain-enriched | |
| **c.2757dup** | 1(0.434782608695652%) | p.Phe920ValfsTer2 | 12 | frameshift |  | P-domain-enriched | |
| **c.2785A>G** | 1(0.434782608695652%) | p.Ile929Val | 12 | missense |  | P-domain-enriched | |
| **c.2804C>T** | 1(0.434782608695652%) | p.Thr935Met | 12 | missense |  | P-domain-enriched | |
| **c.2824del** | 1(0.434782608695652%) | p.Ile942SerfsTer25 | 12 | frameshift |  | P-domain-enriched | |
| **c.2828G>A** | 1(0.434782608695652%) | p.Gly943Asp | 12 | missense |  | P-domain-enriched | |
| **c.2924C>A** | 1(0.434782608695652%) | p.Ser975Tyr | 13 | missense |  | P-domain-enriched | |
| **c.3007G>A** | 1(0.434782608695652%) | p.Ala1003Thr | 13 | missense |  | P-domain-enriched | |
| **c.3044T>C** | 1(0.434782608695652%) | p.Leu1015Pro | 13 | missense |  | P-domain-enriched | |
| **c.3089G>A** | 1(0.434782608695652%) | p.Gly1030Asp | 14 | missense |  | P-domain-enriched | |
| **c.3139del** | 1(0.434782608695652%) | p.Asp1047MetfsTer74 | 14 | frameshift |  | P-domain-enriched | |
| **c.3182G>A** | 1(0.434782608695652%) | p.Gly1061Glu | 14 | missense |  | N-domain-enriched | |
| **c.3188C>T** | 1(0.434782608695652%) | p.Ala1063Val | 14 | missense |  | N-domain-enriched | |
| **c.3305T>C** | 1(0.434782608695652%) | p.Ile1102Thr | 15 | missense |  | N-domain-enriched | |
| **c.3359_3360insTT** | 1(0.434782608695652%) | p.Leu1120fs | 15 | frameshift |  | N-domain-enriched | |
| **c.3577G>C** | 1(0.434782608695652%) | p.Ala1193Pro | 17 | missense |  | N-domain-enriched | |
| **c.3646G>T** | 1(0.434782608695652%) | p.Val1216Leu | 17 | missense |  | N-domain-enriched | |
| **c.3677C>T** | 1(0.434782608695652%) | p.Thr1226Ile | 17 | missense |  | N-domain-enriched | |
| **c.3700delG** | 1(0.434782608695652%) | p.Val1234Leufs*96 | 18 | frameshift |  | N-domain-enriched | |
| **c.3742A>G** | 1(0.434782608695652%) | p.Lys1248Glu | 18 | missense |  | N-domain-enriched | |
| **c.3767_3768insCA** | 1(0.434782608695652%) | p.Gln1256Hisfs*75 | 18 | frameshift |  | Core (post-N) | |

Continued Table S1.ATP7B nucleotide variants present in the children.

| Nucleotide change | Count | Protein change | Exon | Variant type | Splice side | Protein region | |
| --- | --- | --- | --- | --- | --- | --- | --- |
| **c.3818C>A** | 1(0.434782608695652%) | p.Pro1273Gln | 18 | missense |  | Core (post-N) | |
| **c.3859G>A** | 1(0.434782608695652%) | p.Gly1287Ser | 18 | missense |  | Core (post-N) | |
| **c.3884C>T** | 1(0.434782608695652%) | p.Ala1295Val | 18 | missense |  | Core (post-N) | |
| **c.4003G>C** | 1(0.434782608695652%) | p.Gly1335Arg | 19 | missense |  | Core (post-N) | |
| **c.4006delA** | 1(0.434782608695652%) | p.Ile1336fs*57 | 19 | frameshift |  | Core (post-N) | |
| **c.4112T>C** | 1(0.434782608695652%) | p.Leu1371Pro | 20 | missense |  | C-ter tail |  |
| **c.4114C>T** | 1(0.434782608695652%) | p.Gln1372Ter | 20 | nonsense |  | C-ter tail |  |
| **c.4128T>G** | 1(0.434782608695652%) | p.Tyr1376Ter | 21 | nonsense |  | C-ter tail |  |
| **c.51+2T>G** | 1(0.434782608695652%) | splicing | 1 | splicing | donor(+) | NA |  |

Percentages are calculated with the total number of detected pathogenic alleles (n = 230) as the denominator.

Table S2. ATP7B Nucleotide Variant Combinations Identified in Pediatric Wilson's Disease Patients

| **No.** | **Count** | **Variant 1** | | | | **Variant 2** | | | | **Variant 3** | | | | **Variant 4** | | | | | |
| --- | --- | --- | --- | --- | --- | --- | --- | --- | --- | --- | --- | --- | --- | --- | --- | --- | --- | --- | --- |
|  |  | **Nucleotide Change** | **Protein Change** | **Inheritance** | **Zygosity** | **Nucleotide Change** | **Protein Change** | **Inheritance** | **Zygosity** | **Nucleotide Change** | **Protein Change** | **Inheritance** | **Zygosity** | **Nucleotide Change** | **Protein Change** | | **Inheritance** | | **Zygosity** |
| 1 | **4** | c.2304dupC | p.Met769HisfsTer26 | Paternal | het | c.2621C>T | p.Ala874Val | Maternal | het | c.2333G>T | p.Arg778Leu | Maternal | het | c.2310C>G | p.Leu770= | Maternal | | Het | |
| 2 | **4** | c.1708-1G>C | p.? | Maternal | het | c.2975C>T | p.Pro992Leu | Paternal | het | c.1168A>G | p.Ile390Val | Maternal | het | c.-210A>T | p.? | Maternal | | Het | |
| 3 | **4** | c.1708-1G>C | p.? | Paternal | het | c.2975C>T | p.Pro992Leu | Maternal | het | c.-210A>T | p.? | Paternal | het | c.1168A>G | p.Ile390Val | Paternal | | Het | |
| 4 | **3** | c.4005_4006insTTATAATGGGTTGGG | p.Ile1336delinsLeuTer | Maternal | — | c.2333G>T | p.Arg778Leu | Paternal | het | c.2310C>G | p.Leu770= | Paternal | het | — | — | — | | — | |
| 5 | **3** | c.2333G>T | p.Arg778Leu | Paternal | het | c.3532A>G | p.Thr1178Ala | Maternal | het | c.3244-2A>G | splicing | Paternal | het | — | — | — | | — | |
| 6 | **3** | c.2333G>T | p.Arg778Leu | — | het | c.2975C>T | p.Pro992Leu | — | het | c.2310C>G | p.Leu770= | — | het | — | — | — | | — | |
| 7 | **3** | c.2333G>T | p.Arg778Leu | — | het | c.2135G>A | p.Trp712Ter | — | het | c.2310C>G | p.Leu770= | — | het | — | — | — | | — | |
| 8 | **3** | c.3305T>C | p.Ile1102Thr | Paternal | het | c.2333G>T | p.Arg778Leu | Maternal | het | c.2310C>G | p.Leu770= | Maternal | het | — | — | — | | — | |
| 9 | **3** | c.2333G>T | p.Arg778Leu | Paternal | het | c.3532A>G | p.Thr1178Ala | Maternal | het | c.2310C>G | p.Leu770= | Paternal | het | — | — | — | | — | |
| 10 | **3** | c.2333G>T | p.Arg778Leu | Paternal | het | c.3140A>T | p.Asp1047Val | Maternal/Sister | het | c.2310C>G | p.Leu770= | Paternal | het | — | — | — | | — | |
| 11 | **3** | c.2139C>G | p.Tyr713Ter | Paternal | het | c.2333G>T | p.Arg778Leu | Maternal | het | c.2310C>G | p.Leu770= | Maternal | het | — | — | — | | — | |
| 12 | **3** | c.2424_2425insA | p.Gly809ArgfsTer1 | Maternal | het | c.2333G>T | p.Arg778Leu | De novo | het | c.2310C>G | p.Leu770= | De novo | het | — | — | — | | — | |
| 13 | **3** | c.2975C>T | p.Pro992Leu | Maternal | het | c.2333G>T | p.Arg778Leu | Paternal | het | c.2310C>G | p.Leu770= | Paternal | het | — | — | — | | — | |
| 14 | **3** | c.2333G>T | p.Arg778Leu | Maternal | het | c.2975C>T | p.Pro992Leu | Paternal | het | c.2310C>G | p.Leu770= | Maternal | het | — | — | — | | — | |

Continued Table S2. ATP7B Nucleotide Variant Combinations Identified in Pediatric Wilson's Disease Patients

| **No.** | **Count** | **Variant 1** | | | | **Variant 2** | | | | **Variant 3** | | | | **Variant 4** | | | | | |
| --- | --- | --- | --- | --- | --- | --- | --- | --- | --- | --- | --- | --- | --- | --- | --- | --- | --- | --- | --- |
|  |  | **Nucleotide Change** | **Protein Change** | **Inheritance** | **Zygosity** | **Nucleotide Change** | **Protein Change** | **Inheritance** | **Zygosity** | **Nucleotide Change** | **Protein Change** | **Inheritance** | **Zygosity** | **Nucleotide Change** | **Protein Change** | | **Inheritance** | | **Zygosity** |
| 15 | **3** | c.2333G>T | p.Arg778Leu | Maternal | het | c.2620G>C | p.Ala874Pro | Paternal | het | c.2310C>G | p.Leu770= | Maternal | het | — | — | — | | — | |
| 16 | **3** | c.2755C>G | p.Arg919Gly | Paternal | het | c.1708-1G>C | splicing | Maternal | het | c.1168A>G | p.Ile390Val | Maternal | het | — | — | — | | — | |
| 17 | **3** | c.2975C>T | p.Pro992Leu | Paternal | het | c.2333G>T | p.Arg778Leu | Maternal | het | c.2310C>G | p.Leu770= | Maternal | het | — | — | — | | — | |
| 18 | **3** | c.2333G>T | p.Arg778Leu | Maternal | het | c.4114C>T | p.Gln1372Ter | Paternal | het | c.2310C>G | p.Leu770= | Maternal | het | — | — | — | | — | |
| 19 | **3** | c.2333G>T | p.Arg778Leu | Paternal | het | c.3053C>T | p.Ala1018Val | Maternal | het | c.2310C>G | p.Leu770= | Paternal | het | — | — | — | | — | |
| 20 | **3** | c.3884C>T | p.Ala1295Val | — | het | c.2333G>T | p.Arg778Leu | — | het | c.2310C>G | p.Leu770= | — | het | — | — | — | | — | |
| 21 | **2** | c.3809A>G | p.Asn1270Ser | — | het | c.2668G>A | p.Val890Met | — | het | — | — | — | — | — | — | — | | — | |
| 22 | **2** | c.3809A>G | p.Asn1270Ser | Paternal | het | c.2668G>A | p.Val890Met | Maternal | het | — | — | — | — | — | — | — | | — | |
| 23 | **2** | c.2828G>A | p.Gly943Asp | Paternal | het | c.2804C>T | p.Thr935Met | Maternal | het | — | — | — | — | — | — | — | | — | |
| 24 | **2** | c.2975C>T | p.Pro992Leu | Maternal | het | c.1708-1G>C | splicing | Paternal | het | — | — | — | — | — | — | — | | — | |
| 25 | **2** | c.2975C>T | p.Pro992Leu | Paternal | het | c.3089G>A | p.Gly1030Asp | Maternal | het | — | — | — | — | — | — | — | | — | |
| 26 | **2** | c.3053C>T | p.Ala1018Val | Maternal | het | c.2975C>T | p.Pro992Leu | Paternal | het | — | — | — | — | — | — | — | | — | |
| 27 | **2** | c.3443T>C | p.Ile1148Thr | Paternal | het | c.3577G>C | p.Ala1193Pro | Maternal | het | — | — | — | — | — | — | — | | — | |
| 28 | **2** | c.3443T>C | p.Ile1148Thr | Maternal | het | c.4006delA | p.Ile1336fsTer57 | Paternal | het | — | — | — | — | — | — | — | | — | |
| 29 | **2** | c.3767_3768insCA | p.Gln1256HisfsTer75 | Maternal | het | c.3044T>C | p.Leu1015Pro | Paternal | het | — | — | — | — | — | — | — | | — | |
| 30 | **2** | c.3182G>A | p.Gly1061Glu | Maternal | het | c.2333G>T | p.Arg778Leu | Paternal | het | — | — | — | — | — | — | — | | — | |

Continued Table S2. ATP7B Nucleotide Variant Combinations Identified in Pediatric Wilson's Disease Patients

| **No.** | **Count** | **Variant 1** | | | | **Variant 2** | | | | **Variant 3** | | | | **Variant 4** | | | | | |
| --- | --- | --- | --- | --- | --- | --- | --- | --- | --- | --- | --- | --- | --- | --- | --- | --- | --- | --- | --- |
|  |  | **Nucleotide Change** | **Protein Change** | **Inheritance** | **Zygosity** | **Nucleotide Change** | **Protein Change** | **Inheritance** | **Zygosity** | **Nucleotide Change** | **Protein Change** | **Inheritance** | **Zygosity** | **Nucleotide Change** | **Protein Change** | | **Inheritance** | | **Zygosity** |
| 31 | **2** | c.3889G>A | p.Val1297Ile | Maternal | het | c.1614-4C>T | splicing | Paternal | het | — | — | — | — | — | — | — | | — | |
| 32 | **2** | c.1470C>A | p.Cys490Ter | Maternal | het | c.2975C>T | p.Pro992Leu | Paternal | het | — | — | — | — | — | — | — | | — | |
| 33 | **2** | c.3359_3360insTT | p.Leu1120fs | Paternal | het | c.2975C>T | p.Pro992Leu | Maternal | het | — | — | — | — | — | — | — | | — | |
| 34 | **2** | c.3532A>G | p.Thr1178Ala | Paternal | het | c.2662A>C | p.Thr888Pro | Maternal | het | — | — | — | — | — | — | — | | — | |
| 35 | **2** | c.3809A>G | p.Asn1270Ser | Maternal | het | c.3562C>T | p.Leu1188Phe | Paternal | het | — | — | — | — | — | — | — | | — | |
| 36 | **2** | c.2755C>G | p.Arg919Gly | Paternal/Sister | het | c.2697_2723del | p.Ile899_Gln907del | Maternal | het | — | — | — | — | — | — | — | | — | |
| 37 | **2** | c.1470C>A | p.Cys490Ter | — | het | c.2662A>C | p.Thr888Pro | — | het | — | — | — | — | — | — | — | | — | |
| 38 | **2** | c.1543+1G>T | p.? | — | het | c.3007G>A | p.Ala1003Thr | Paternal | het | — | — | — | — | — | — | — | | — | |
| 39 | **2** | c.3809A>G | p.Asn1270Ser | Maternal | het | c.3140A>T | p.Asp1047Val | Paternal | het | — | — | — | — | — | — | — | | — | |
| 40 | **2** | c.2333G>T | p.Arg778Leu | Biparental | hom | c.2310C>G | p.Leu770= | Biparental | hom | — | — | — | — | — | — | — | | — | |
| 41 | **2** | c.2333G>T | p.Arg778Leu | Biparental | hom | c.2310C>G | p.Leu770= | Biparental | hom | — | — | — | — | — | — | — | | — | |
| 42 | **2** | c.2333G>T | p.Arg778Leu | — | hom | c.2310C>G | p.Leu770= | — | hom | — | — | — | — | — | — | — | | — | |
| 43 | **2** | c.1543+1G>T | p.? | — | het | c.3562C>T | p.Leu1188Phe | — | het | — | — | — | — | — | — | — | | — | |
| 44 | **2** | c.3809A>G | p.Asn1270Ser | — | het | c.3532A>G | p.Thr1178Ala | — | het | — | — | — | — | — | — | — | | — | |
| 45 | **2** | c.2975C>T | p.Pro992Leu | Paternal | het | c.3443T>C | p.Ile1148Thr | Maternal | het | — | — | — | — | — | — | — | | — | |
| 46 | **2** | c.2975C>T | p.Pro992Leu | — | het | c.2668G>A | p.Val890Met | — | het | — | — | — | — | — | — | — | | — | |

Continued Table S2. ATP7B Nucleotide Variant Combinations Identified in Pediatric Wilson's Disease Patients

| **No.** | **Count** | **Variant 1** | | | | **Variant 2** | | | | **Variant 3** | | | | **Variant 4** | | | | | |
| --- | --- | --- | --- | --- | --- | --- | --- | --- | --- | --- | --- | --- | --- | --- | --- | --- | --- | --- | --- |
|  |  | **Nucleotide Change** | **Protein Change** | **Inheritance** | **Zygosity** | **Nucleotide Change** | **Protein Change** | **Inheritance** | **Zygosity** | **Nucleotide Change** | **Protein Change** | **Inheritance** | **Zygosity** | **Nucleotide Change** | **Protein Change** | | **Inheritance** | | **Zygosity** |
| 47 | **2** | c.2662A>C | p.Thr888Pro | Paternal | het | c.2333G>T | p.Arg778Leu | Maternal | het | — | — | — | — | — | — | — | | — | |
| 48 | **2** | c.2304dup | p.Met769HisfsTer26 | Paternal | het | c.3443T>C | p.Ile1148Thr | Maternal | het | — | — | — | — | — | — | — | | — | |
| 49 | **2** | c.1543+1G>T | p.? | — | het | c.2662A>C | p.Thr888Pro | — | het | — | — | — | — | — | — | — | | — | |
| 50 | **2** | c.2975C>T | p.Pro992Leu | Maternal | het | c.2621C>T | p.Ala874Val | Paternal | het | — | — | — | — | — | — | — | | — | |
| 51 | **2** | c.2038C>T | p.Gln680Ter | Maternal | het | c.3532A>G | p.Thr1178Ala | Paternal | het | — | — | — | — | — | — | — | | — | |
| 52 | **2** | c.3443T>C | p.Ile1148Thr | Paternal | het | c.2447+5G>T | splicing | Maternal | het | — | — | — | — | — | — | — | | — | |
| 53 | **2** | c.3244-2A>G | splicing | Maternal | het | c.3446G>A | p.Gly1149Glu | Paternal | het | — | — | — | — | — | — | — | | — | |
| 54 | **2** | c.1470C>A | p.Cys490Ter | Maternal | het | c.3646G>A | p.Val1216Met | Maternal | het | — | — | — | — | — | — | — | | — | |
| 55 | **2** | c.2078C>G | p.Ser693Cys | Maternal | het | c.2975C>T | p.Pro992Leu | Paternal | het | — | — | — | — | — | — | — | | — | |
| 56 | **2** | c.2621C>T | p.Ala874Val | Maternal | het | c.3646G>A | p.Val1216Met | Maternal | het | — | — | — | — | — | — | — | | — | |
| 57 | **2** | c.3766_3767dup | p.Gln1256HisfsTer75 | Paternal/Sister | het | c.3677C>T | p.Thr1226Ile | Maternal/Sister | het | — | — | — | — | — | — | — | | — | |
| 58 | **2** | c.525dup | p.Val176SerfsTer28 | Paternal | het | c.2975C>T | p.Pro992Leu | Maternal | het | — | — | — | — | — | — | — | | — | |
| 59 | **2** | c.4003G>C | p.Gly1335Arg | Paternal | het | c.2975C>T | p.Pro992Leu | Maternal | het | — | — | — | — | — | — | — | | — | |
| 60 | **2** | c.2621C>T | p.Ala874Val | Maternal | het | c.1708-5T>G | splicing | Paternal | het | — | — | — | — | — | — | — | | — | |
| 61 | **2** | c.2975C>T | p.Pro992Leu | Maternal | het | c.2333G>T | p.Arg778Leu | Paternal | het | — | — | — | — | — | — | — | | — | |

Continued Table S2. ATP7B Nucleotide Variant Combinations Identified in Pediatric Wilson's Disease Patients

| **No.** | **Count** | **Variant 1** | | | | **Variant 2** | | | | **Variant 3** | | | | **Variant 4** | | | | | |
| --- | --- | --- | --- | --- | --- | --- | --- | --- | --- | --- | --- | --- | --- | --- | --- | --- | --- | --- | --- |
|  |  | **Nucleotide Change** | **Protein Change** | **Inheritance** | **Zygosity** | **Nucleotide Change** | **Protein Change** | **Inheritance** | **Zygosity** | **Nucleotide Change** | **Protein Change** | **Inheritance** | **Zygosity** | **Nucleotide Change** | **Protein Change** | | **Inheritance** | | **Zygosity** |
| 62 | **2** | c.2975C>T | p.Pro992Leu | Paternal | het | c.3960G>C | p.Arg1320Ser | Maternal | het | — | — | — | — | — | — | — | | — | |
| 63 | **2** | c.525dupA | p.Val176SerfsTer28 | Maternal | het | c.2755C>G | p.Arg919Gly | Paternal | het | — | — | — | — | — | — | — | | — | |
| 64 | **2** | c.3443T>C | p.Ile1148Thr | Paternal | het | c.3139del | p.Asp1047MetfsTer74 | Maternal | het | — | — | — | — | — | — | — | | — | |
| 65 | **2** | c.3818C>A | p.Pro1273Gln | Maternal | het | c.3742A>G | p.Lys1248Glu | Paternal | het | — | — | — | — | — | — | — | | — | |
| 66 | **2** | c.3766_3767dupCA | p.Gln1256HisfsTer75 | Maternal | het | c.4128T>G | p.Tyr1376Ter | Paternal | het | — | — | — | — | — | — | — | | — | |
| 67 | **2** | c.3446G>A | p.Gly1149Glu | Paternal | het | c.1708-5T>G | splicing | Maternal | het | — | — | — | — | — | — | — | | — | |
| 68 | **2** | c.2195+5G>T | splicing | Maternal | het | c.2333G>T | p.Arg778Leu | Paternal | het | — | — | — | — | — | — | — | | — | |
| 69 | **2** | c.2333G>T | p.Arg778Leu | Paternal | het | c.3646G>A | p.Val1216Met | Maternal/Brother | het | — | — | — | — | — | — | — | | — | |
| 70 | **2** | c.3443T>C | p.Ile1148Thr | — | het | c.2757dup | p.Phe920ValfsTer2 | — | het | — | — | — | — | — | — | — | | — | |
| 71 | **2** | c.2304dup | p.Met769HisfsTer26 | Paternal | het | c.3646G>A | p.Val1216Met | Maternal | het | — | — | — | — | — | — | — | | — | |
| 72 | **2** | c.51+2T>G | splicing | Maternal | het | c.4112T>C | p.Leu1371Pro | Paternal | het | — | — | — | — | — | — | — | | — | |
| 73 | **2** | c.2333G>T | p.Arg778Leu | Biparental | hom | c.2310C>G | p.Leu770= | Biparental | hom | — | — | — | — | — | — | — | | — | |
| 74 | **2** | c.3446G>A | p.Gly1149Glu | Maternal | het | c.2755C>G | p.Arg919Gly | Paternal | het | — | — | — | — | — | — | — | | — | |

Continued Table S2. ATP7B Nucleotide Variant Combinations Identified in Pediatric Wilson's Disease Patients

| **No.** | **Count** | **Variant 1** | | | | **Variant 2** | | | | **Variant 3** | | | | **Variant 4** | | | | | |
| --- | --- | --- | --- | --- | --- | --- | --- | --- | --- | --- | --- | --- | --- | --- | --- | --- | --- | --- | --- |
|  |  | **Nucleotide Change** | **Protein Change** | **Inheritance** | **Zygosity** | **Nucleotide Change** | **Protein Change** | **Inheritance** | **Zygosity** | **Nucleotide Change** | **Protein Change** | **Inheritance** | **Zygosity** | **Nucleotide Change** | **Protein Change** | | **Inheritance** | | **Zygosity** |
| 75 | **2** | c.3700delG | p.Val1234LeufsTer96 | Paternal | het | c.3960G>C | p.Arg1320Ser | Maternal | het | — | — | — | — | — | — | — | | — | |
| 76 | **2** | c.2621C>T | p.Ala874Val | Paternal | het | c.3809A>G | p.Asn1270Ser | Maternal | het | — | — | — | — | — | — | — | | — | |
| 77 | **1** | c.2975C>T | p.Pro992Leu | Paternal | hom | — | — | — | — | — | — | — | — | — | — | — | | — | |
| 78 | **1** | c.2975C>T | p.Pro992Leu | Biparental | hom | — | — | — | — | — | — | — | — | — | — | — | | — | |
| 79 | **1** | c.2333G>T | p.Arg778Leu | Biparental | hom | — | — | — | — | — | — | — | — | — | — | — | | — | |
| 80 | **1** | c.2975C>T | p.Pro992Leu | — | hom | — | — | — | — | — | — | — | — | — | — | — | | — | |
| 81 | **1** | c.2785A>G | p.Ile929Val | — | hom | — | — | — | — | — | — | — | — | — | — | — | | — | |
| Abbreviations: het, het; hom, hom. Inheritance denotes the parent confirmed to carry the respective variant by Sanger sequencing. De novo indicates variants identified in the proband without parental carrier confirmation. —, not applicable or not determined. | | | | | | | | | | | | | | | | | | | |
